# Environmental and Lifestyle Risk Factors in the Carcinogenesis of Gallbladder Cancer

**DOI:** 10.3390/jpm12020234

**Published:** 2022-02-08

**Authors:** Pablo Pérez-Moreno, Ismael Riquelme, Patricia García, Priscilla Brebi, Juan Carlos Roa

**Affiliations:** 1 Millennium Institute on Immunology and Immunotherapy, Department of Pathology, School of Medicine, Pontificia Universidad Católica de Chile, Santiago 8380000, Chile; pablo.perezm@uc.cl (P.P.-M.); pgarciam@uc.cl (P.G.); 2 Institute of Biomedical Sciences, Faculty of Health Sciences, Universidad Autónoma de Chile, Temuco 4810101, Chile; ismael.riquelme@uautonoma.cl; 3 Millennium Institute on Immunology and Immunotherapy, Laboratory of Integrative Biology (LiBi), Centro de Excelencia en Medicina Translacional (CEMT), Scientific and Technological Bioresource Nucleus (BIOREN), Universidad de La Frontera, Temuco 4810296, Chile; priscilla.brebi@ufrontera.cl

**Keywords:** gallbladder cancer, risk factors, carcinogenesis

## Abstract

Gallbladder cancer (GBC) is an aggressive neoplasm that in an early stage is generally asymptomatic and, in most cases, is diagnosed in advanced stages with a very low life expectancy because there is no curative treatment. Therefore, understanding the early carcinogenic mechanisms of this pathology is crucial to proposing preventive strategies for this cancer. The main risk factor is the presence of gallstones, which are associated with some environmental factors such as a sedentary lifestyle and a high-fat diet. Other risk factors such as autoimmune disorders and bacterial, parasitic and fungal infections have also been described. All these factors can generate a long-term inflammatory state characterized by the persistent activation of the immune system, the frequent release of pro-inflammatory cytokines, and the constant production of reactive oxygen species that result in a chronic damage/repair cycle, subsequently inducing the loss of the normal architecture of the gallbladder mucosa that leads to the development of GBC. This review addresses how the different risk factors could promote a chronic inflammatory state essential to the development of gallbladder carcinogenesis, which will make it possible to define some strategies such as anti-inflammatory drugs or public health proposals in the prevention of GBC.

## 1. Introduction

Gallbladder cancer (GBC) is a neoplasm that causes around 84,695 deaths per year worldwide [1], affecting more commonly women than men with an age-standardized incidence rate of 1.4 and 0.89 per 100,000 people, respectively [1]. The onset of this disease is characterized by a slow and silent development, resulting in many GBC cases being frequently diagnosed at late stages when patients have a 5-year life expectancy of less than 5% [2]. For this reason, the understanding of how different risk factors influence the early development of gallbladder carcinogenic processes is crucial to improving the prevention strategies for this malignancy.

GBC has multifactorial causes that converge to form a new tumor. The most important risk factor described for GBC development is gallstone disease (GSD) due to its high correlation with GBC [3,4,5,6]. Gallstones can lead to a chronic inflammatory state of tissue damage/regeneration characterized by an imbalance between anti- and pro-inflammatory factors that induce carcinogenic processes in the gallbladder mucosa [6]. In fact, some environmental and lifestyle risk factors may be involved in the development of gallstones and the subsequent GBC formation, such as body mass index (BMI), geographical location, nutritional aspects (high-fat and high-sugar diets), bacterial infections (e.g., *Salmonella typhi*), chronic diseases (e.g., sclerosing cholangitis, type II diabetes, metabolic syndrome and/or dyslipidemias), parasitic infections, smoking, and alcohol consumption [2,7,8,9,10,11,12,13,14,15,16].

Therefore, this article reviews the relationship between environmental risk factors and the formation of chronic inflammation, and how this inflammatory process is involved in gallbladder carcinogenesis.

## 2. Search and Selection of Literature

**Figure 1:**
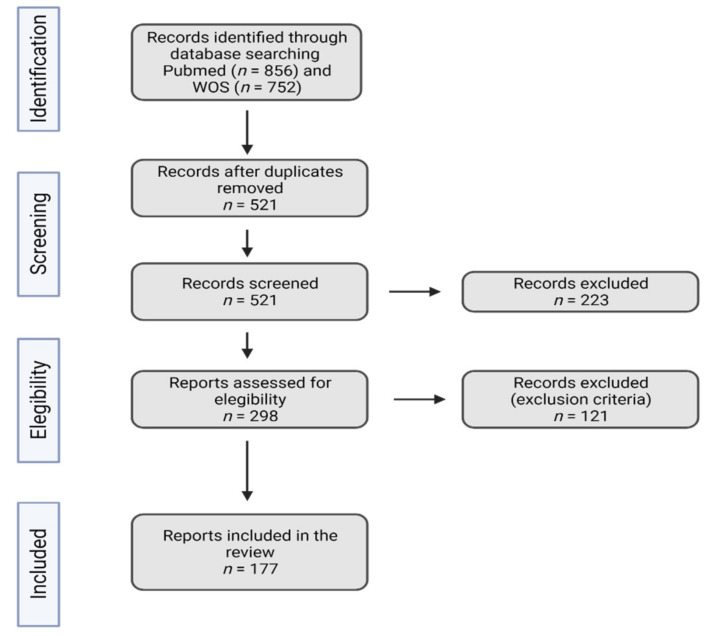
Diagram used in the systematic review.

A literature search was performed using different databases including Pubmed and Web of Science. The search terms were as follows: (gallbladder cancer OR gallbladder neoplasia OR gallbladder carcinoma) AND (inflammation OR environmental risk factors OR carcinogenesis OR lifestyle OR bacterial infections OR viral infections OR parasitic infections OR fungal infections OR immune diseases OR metabolic diseases OR alcohol OR smoking OR aflatoxins). The selection criteria of articles were as follows: (1) articles related to risk factors associated with gallbladder cancer and (2) articles related to inflammation in gallbladder cancer. Those studies that were not related to GBC were excluded from this review. In those cases of multiple publications regarding the same topic, we consider only the most relevant studies. We identified one retrospective [17], five prospective [15,16,18,19,20], nine meta-analyses [9,13,14,21,22,23,24,25,26], eleven case–control [3,4,27,28,29,30,31,32,33,34], and two Mendelian randomization [35,36] studies. Other additional sources of information consisted of experimental studies and review articles. The search diagram is represented in Figure 1.

The selection criteria of articles were: (i) articles related to risk factors associated with gallbladder cancer and (ii) articles related to inflammation in gallbladder cancer. The exclusion criteria used in the screening phase focused on those studies that were not related to GBC; meanwhile, in the eligibility phase, the exclusion criteria were focused on those studies regarding the same topic.

## 3. Inflammation in Gallbladder Carcinogenesis

Inflammation is a process that involves the activation of the innate and adaptive immune system to defend the host against pathogens, as well as to repair and remodel tissues to recover their homeostasis [37]. Thus, the main physiological role of inflammation is to be activated after foreign damage that occurs in a specific area of the body, eliminating the Noxa and allowing the restoration of homeostasis in the affected area [37,38]. Under normal conditions this process begins with vasodilation and hyperemia within the blood vessels that promote vascular permeability, mainly induced by the increase in nitric oxide (NO) and allowing the immune cells and inflammatory mediators (the inflammatory exudate) to exit towards the extravascular tissues. This induces many circulating leukocytes to be able to adhere to the endothelium through molecules, such as E-selectin and VCAM-1, located on the surface of activated endothelial cells. Then, the monocytes are transformed into macrophages that release many hydrolytic enzymes, pro-inflammatory cytokines such as IL-1 and TNF-α, and free radicals that result in the development of an acute inflammatory process in epithelial cells. Finally, when the injuring agent has disappeared, macrophages stimulate fibroblasts for collagen synthesis and endothelial cells to produce growth factors that stimulate tissue repair. Fortunately, under normal conditions, this process is strictly regulated [37,39]. However, in a pathological context such as cancer, this process occurs chronically, allowing a vicious cycle of damage/repair, which leads to chronic DNA damage, generating mutations in different genes, which can promote the development of cancer [37,39,40,41].

Unlike what occurs with a normal inflammatory response, in cancer there is a hyperproliferation of epithelial cells destined to increase the number of macrophages able to react against tumor cells, which acquire different protein expression patterns and phenotypical changes that lead to being recognized as foreign to the host [41,42]. In addition, after an oncogenic injury, the immune response will not be able to eliminate the Noxa (oncogenic injury); thus, an increase in the inflammatory reaction will be observed, resulting in a higher tumor growth rather than a restoration of tissue homeostasis [42]. Another difference between normal inflammation and cancer-inducing inflammation is that in a normal inflammation context against harmful agents (e.g., bacterial infections), the inflammatory response has an acute character by inducing the recruitment of macrophages from the nearby tissues and the diapedesis of monocytes from nearby blood vessels to be transformed into macrophages in the corresponding tissue. Conversely, due to chronic inflammation during the pre-carcinogenic stages, cancer is commonly characterized by a local proliferation of macrophages and migration of more macrophages from nearby sites to the affected area, which would indicate a weaker immune system activation. However, this effect takes longer and could increase as the tumor grows [42,43,44]. Interestingly, about 15% of cancers—including GBC—are preceded by chronic inflammation, which can be local (e.g., inflammation due to gallstones or infections) or systemic (e.g., metabolic syndrome) [45,46]. Therefore, the inflammatory component of GBC can come from different sources, producing an inflammatory state of a summative nature that provides the ideal niche for carcinogenic progress.

As mentioned above, chronic inflammation is widely described as a crucial early factor in the development of cancer within different organs [39,47]. One of the most studied cytokines that has been linked to cancer is tumor necrosis factor α (TNF-α) [48]. It has been shown that cancer cells release TNF-α, promoting cell transformation, tumorigenesis, and metastasis [48]. Surprisingly, TNF-α expression increases from hyperplasia to carcinoma, and also during TNM stages in GBC tissues [49], suggesting that TNF-α could participate in carcinogenic processes and may be a progression marker in GBC patients. Other cytokines are IL-2 and IL-8, which have been shown to promote COX-2 expression, which is overexpressed in chronic cholecystitis, increasing their expression in GBC tissues [50], suggesting that metabolites produced via cyclooxygenases (e.g., arachidonic acid) mediate the inflammatory response of the gallbladder [51]. Interestingly, the pain produced by gallstones is treated with non-steroidal anti-inflammatory drugs (NSAIDs), inhibiting the activity of COX-2 [52,53]. In addition, the COX-2 protein is intensively expressed in the hyperplastic mucosa of the gallbladder in patients with an anomalous arrangement of the pancreaticobiliary duct (AAPBD) [54], a congenital malformation in which the pancreatic and bile ducts join outside the wall of the duodenum, allowing pancreatic juices to chronically damage the bile duct causing an increased risk of developing GBC [55,56]. Interestingly, about 61% of patients with AAPBD have hyperplasia in the gallbladder mucosa [57,58], suggesting that the function and expression of COX-2 are increased from early stages in GBC carcinogenesis, participating in the malignant transformation of gallbladder mucosal cells. In addition, the prevalence of KRAS mutations is higher in AAPBD-related GBC [59], providing evidence that mutations in KRAS are involved in gallbladder carcinogenesis [60,61,62].

In 1986, Yamagiwa et al. proposed a GBC progression sequence through which a normal mucosa develops metaplasia, then dysplasia and ultimately a carcinoma [63]. It has been found that some common genetic markers such as *KRAS* [64,65], *PIK3CA* [64], and *HER2/neu* (c-Erbb-2) [66] are altered in different progression stages of this disease through loss of heterozygosity (LOH) and changes in the DNA methylation [67]. Accordingly, different genes have shown a deregulated expression in the early stages of GBC [68,69,70,71]. Recently, a study by Brägelmann et al. [72] showed different epigenetic changes that occur during the different stages of GBC carcinogenesis (i.e., GSD -> dysplasia -> GBC). For in-stance, epigenetic changes were observed to be accentuated in different genes as the tumor progression advanced, highlighting the hypermethylation of cytosine-guanine dinucleotide islands (CpG) and gene promoter regions. The results showed that methylation occurred in genes involved in the control of certain signaling pathways such as Wnt, an important pathway that is overactive during the carcinogenic process of different malignancies, including GBC. Among the differentially methylated regions were found the promoter regions of *ZIC1, HHIP* and *PTCH1* (two negative regulators of Hedgehog signaling), *WIF1, RUNX3, P73, RPRM, TWIST1, HBE1*, and *DCLK1*, among other genes. In addition, gene promoters with hypomethylation were found in *HMGA1, ERBB2, CDCA7*, and *RUNX1,* among others. Furthermore, higher copy numbers were frequently found in some proto-oncogenes such as MDM2 and YEATS4, which suggest that these findings could have a real impact on GBC carcinogenesis and malignancy [72]. In addition, alterations in the TP53 gene have been observed in tissues from patients with chronic cholecystitis [69,73]. Another example is the case of the fragile histidine triad (FHIT) gene, which encodes an enzyme involved in purine metabolism that commonly sees a 55% loss of expression and a 46% allelic loss in dysplastic lesions in GBC patients [74]. In a study by Roa et al. [75], some somatic mutations in *PI3KCA* were found in the tissues of early GBC cases. Similarly, a study by Li et al. demonstrated that PI3K was overexpressed in gallbladder polyps and even higher in GBC tissues [76]. Interestingly, PI3K actively participates in the regulation of the immune response [77], suggesting that early alterations in PI3K could drastically alter the balance of the inflammatory response. Furthermore, Espinoza et al. also described Claudin-18 (CLDN-18) as a membrane marker of gallbladder metaplasia within the progression sequence towards GBC, where the CLDN-18 expression is also present in ~50% of gallbladder tumors [78].

A study by Mishra et al. [79] indicated that certain mutations in IDH1, IDH2, and KMT2C genes could generate a greater susceptibility to developing GBC. Additionally, Salazar et al. addressed the alteration of other important genes that could also be involved in gallbladder carcinogenesis, including *Rb, VHL, EGFR, MSI*, and *hTERT* [80]. Similarly, a study by Pandey et al. showed that some cholecystitis samples had alterations in the *PIK3R2*, *CHD1*, *TP53,* and *CDKN2A* genes. Interestingly, these alterations were also found in GBC samples, suggesting that these mutations are present from the early stages of gallbladder carcinogenesis towards the establishment of carcinoma. In addition, these authors identified other significantly mutated genes in GBC, including *CCTNNB1*, *ELF3*, *ERBB2*, *ARID2*, *ERBB3*, *STK11*, *SMAD4*, *ARID1A*, *KRAS*, *EHF*, *PIK3CA*, *BRAF*, *ACVR2A*, *PSIP1*, *NFE2L2*, *CHRM3*, *ZNF107*, *SMARCA4*, *APC*, *NF1*, *KAT8*, *MAP2K4,* and *HIST1H2AG*. In particular, mutations in the *TP53*, *ELF3*, *CTNNB1*, *ERBB2*, *ARID1A,* and *CDKN2A* genes resulted in the formation of different peptides as neoantigens. Among these genes, ELF3 had the highest number (*n* = 9) of predicted neoantigens capable of inducing T-cell activation and, therefore, these neoantigens may become candidates for GBC vaccines [81].

All these results suggest that the mutations and aberrant expression of these genes could be responsible for promoting gallbladder carcinogenesis [82].

Alterations in these genes have also been found in the carcinogenic sequences of other cancer types. For instance, different types of *TP53* mutations have been described in the early preneoplastic stages of gastric, colorectal, and lung cancers [83,84,85,86], strongly suggesting that *TP53* is altered in the initial stages of cancer probably induced by early and persistent inflammatory processes.

As suggested by the different studies reviewed above, alterations in the *TP53* gene could be a crucial event for the beginning of a carcinogenic process. The main consequence of *TP53* alterations is the loss of its function, decreasing its capacity as a tumor suppressor, causing cells to begin to lose regulation of the cell cycle, and perpetuating the successive mutations in DNA that translate into greater genomic instability [87]. In this regard, the frequency of TP53 abnormalities observed in early stages, such as chronic cholecystitis, can be subgrouped into 35% of LOH, 25% of mutations, and 11% of gene inactivation; meanwhile, in invasive GBC the frequency is 81%, 67 %, and 52%, respectively. This suggests that alterations in TP53 increase dramatically as disease progression increases [69]. From the point of view of the inflammatory process, TP53 abnormalities are significant since one of their functions is to repress the inflammatory response mainly through the NF-κb pathway [88]. The NF-κb pathway has been described as a key regulator of pro-inflammatory response in cancer progression [89,90] by promoting the expression of certain cytokines (e.g., IL-1, IL-2, IL-6, and TNF-α), some chemokines (e.g., CXCL1, CXCL10, and MCP-1), adhesion molecules (ICAM-1, VCAM-1, and ECAM-1), and antiapoptotic factors such as BCL-2, c-Flip, and survivin, allowing the recruitment and activation of leukocytes and tumor cell survival [91,92]. Therefore, all these previously described factors suggest that the loss of p53 function in GBC could be an important cause in the imbalance of the early inflammatory response and, as a consequence, in gallbladder carcinogenesis.

## 4. Nutritional and Lifestyle Aspects That Increase Susceptibility to Gallbladder Carcinogenesis

As previously mentioned, gallstone disease (GSD) is one of the most important predisposing factors to developing GBC. Thus, metabolic syndrome and some factors that increase the probability of forming gallstones, including sedentary lifestyle, the consumption of sugar-sweetened and artificially sweetened beverages, obesity, high-fat diet, hypercholesterolemia, and the consumption of red meat, can consequently promote carcinogenesis by inducing a chronic pro-inflammatory state in the gallbladder tissue [2,6,27,93]. Conversely, a physically active lifestyle and the consumption of some vegetables, such as radish and sweet potato, have been shown to reduce the risk of developing gallstones and GBC [27,94]. In this regard, an increased BMI constitutes an important risk factor for GBC and other types of cancer [21,95]. For instance, a study by Barahona et al. showed that BMI has a causal effect on gallstone disease, which subsequently increases the GBC risk. The meta-analysis performed by Tan et al. showed that the relative risk of GBC was 1.14 (95% CI, 1.04–1.25) for overweight people (BMI 25–30 kg/m^2^) and 1.56 (95% CI, 1.41–1.73) for obese individuals (BMI > 30 kg/m^2^), evidencing a higher GBC risk in women than men [22]. This difference between genders could be explained by the fact that estrogens promote a greater cholesterol storage in the bile, which is consistent with the higher obesity and BMI rates observed in women, and the increased risk of developing gallstones [96]. Interestingly, higher C-reactive protein (CRP) concentrations increased GBC risk in the European population, suggesting that the inflammatory status is crucial in developing GBC [36].

The matter of a sedentary lifestyle can be explained according to metabolic disorders inferred from the study by Skoumas et al., in which physically active women had significantly lower levels of total serum cholesterol, LDL-c, oxidized LDL cholesterol, and triglycerides compared to sedentary women [94]. Other studies have also observed that a more sedentary lifestyle is directly correlated with the risk of developing metabolic syndrome and elevated plasma levels of triglycerides and cholesterol [97,98]. On the other hand, the consumption of sugar-sweetened and artificially sweetened beverages can significantly increase (double) the risk of GBC when individuals consume two or more servings per day (200 mL/serving) of sweetened beverages compared to those without consumption (hazard ratio = 2.24; 95% CI = 1.02 to 4.89) [18]. A probable explanation for this is that the increased sugar intake has been associated with increased body weight and diabetes mellitus, predisposing to gallstone disease-dependent GBC [19]. In fact, diabetic patients have shown a higher risk of GBC compared to non-diabetic individuals [13]. This phenomenon is probably due to the fact that hyperinsulinemia caused by sugar ingestion induces the expression of the insulin-like growth factor receptor (IGFR-1), which is able to induce malignant properties in several types of cancer by increasing cell proliferation, tumorigenic capacity, and apoptosis resistance through the activation of PI3K and MAPK pathways [99].

In general, these data suggest that a sedentary lifestyle and sugar consumption—powered by high-fat food—can promote gallstone formation and subsequently GBC.

**Figure 2:**
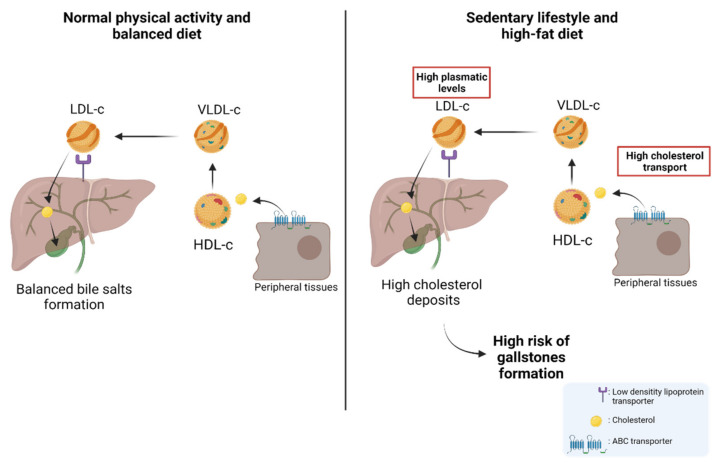
The cholesterol metabolism and the risk of developing gallstones. Left: Under normal conditions, cholesterol stored in peripheral tissues is transported to HDL-c via ABC transporters. The circulating HDL-c in the blood transports cholesterol to VLDL-c which is then transformed into LDL-c. Then, LDL-c transports cholesterol to the hepatocytes through a low-density lipoprotein receptor (LDLr). Cholesterol can be used in bile formation and stored in the gallbladder. Right: As a result of a high-fat diet and sedentary lifestyle, the concentration of plasma cholesterol increases, provoking a greater presence of cholesterol in peripheral tissues. This causes increased transportation of cholesterol from peripheral tissues to the liver via the different lipoproteins (HDL-c, VLDL-c, and LDL-c). Finally, this induces an increase in the storage of cholesterol in the liver and subsequently a greater release of cholesterol from the liver to the gallbladder, which leads to a high risk of gallstone formation.

About 80% of gallstones are comprised of cholesterol; hence, patients with permanently high levels of plasma cholesterol are candidates for gallstones [100,101,102,103]. Physiologically, cholesterol is transported through different lipoproteins such as high-density lipoproteins (HDL-c) and low-density lipoproteins (LDL-c). HDL-c takes the cholesterol from peripheral tissues through ABC transporters (e.g., ABCA1) and esterifies it through lecithin cholesterol acyltransferase (LCAT) [104]. Subsequently, HDL-c can be recognized by the scavenger receptor class B type I (SR-BI), which facilitates the uptake of high-density lipoprotein cholesterol esters in the liver. In addition, cholesterol can be transported to very-low-density lipoproteins (VLDL-c) via the cholesteryl ester transfer protein (CETP) [105,106]. Then, VLDL-c is transformed into LDL-c and is recognized by the low-density lipoprotein receptor (LDLr) and LDL-related protein (LRP) to induce the internalization of cholesterol into the hepatocytes in the liver [105,107]. Finally, the cholesterol within the hepatocytes can be used in bile formation to be transported to the bile canaliculus and stored in the gallbladder [105,108]. Interestingly, a study by Selcuk Atamanalp et al. showed that high cholesterol and LDL-c levels in plasma were closely correlated with a higher presence of cholesterol gallstones. Conversely, low serum HDL levels did not affect the occurrence of cholesterol gallstones [109]. In addition, Wang et al. showed that patients with increased plasma levels of cholesterol, triglycerides, HDL-c, LDL-c, and apolipoprotein B (APOB), also showed a significantly higher recurrence of gallstones compared to control patients [110], which was similar to those results found by Hayat et al., who showed that patients with gallstones had significantly higher plasma levels of triglycerides and HDL-c than the control patients [20]. All these data reaffirm the idea that high plasma levels of cholesterol and other lipids induce gallstone formation and, therefore, are heavily involved in the subsequent development of GBC. The cholesterol metabolism and its implication in the risk of developing GSD are represented in Figure 2.

There are different pathophysiological processes that promote gallstone formation, including cholesterol crystallization/nucleation, alteration in mucin secretion, changes in biliary motility, alteration in intestinal cholesterol transport, and intestinal motility [93]. Normally, cholesterol is found in unilamellar vesicles that bind bile salts to allow cholesterol solubilization. However, when there is an increase in cholesterol deposits, the solubilizing capacity of the bile is saturated promoting the solidification and subsequent nucleation of cholesterol/bile acids, allowing the formation of gallstones [111]. The mechanism by which cholelithiasis predisposes to GBC has not yet been established; however, it has been reported that the DNA mutation rate is higher in the inflammation microenvironment induced by gallstones than in normal tissues, increasing the risk of developing GBC [112]. This inflammatory state is generated in different ways, including the direct damage by gallstones due to continuous friction with the mucosa, which leads to a continuous damage/repair loop in the gallbladder epithelium and, on the other hand, the increase in gallstone size which can obstruct the bile duct, which also increases the susceptibility to infections that give rise to the inflammation process [93,113]. 

Different animal models have been used to study gallstone formation and its effect on the development of preneoplastic lesions [114,115,116,117]. Our research group established an animal model of gallbladder preneoplasia through a lithogenic diet. The results showed that after administration of a high-cholesterol diet (lithogenic diet) for 9 months, early gallstone formation was induced within the gallbladder. Furthermore, animals fed a lithogenic diet evidenced fatty liver and higher plasma cholesterol levels than animals fed a normal diet in a similar manner to that observed in humans. More interestingly, those animals treated with a lithogenic diet had metaplastic and dysplastic architecture in gallbladder tissues, with no invasive features, but with a well-defined inflammatory component showing a predominance of lymphocytes and polymorphonuclear cells [116]. A similar study showed that mice treated with a lithogenic diet had epithelial hyperplasia along with the occurrence of acute and chronic inflammation characterized by the presence of eosinophils, neutrophils, and lymphocytes within the lamina propria [117]. Recently, Kato et al. described an animal model generated by the orthotopic implantation of gallbladder organoids containing mutant loss of *KRAS* and *TP53* genes developed in vitro using lentiviral Cre transduction and CRISPR/Cas9 gene editing, respectively. The data showed that the tumor transcriptomic profiles are similar to that found in human tissues, as well as the immune cell infiltration observed during tumor formation, suggesting that this model could be an interesting approach to study carcinogenesis in GBC [118].

Different inflammatory components have been described as participants in the pathophysiology of inflammation including some inflammatory cytokines such as IL-6, IL-10, IL-12, and visfatin. Interestingly, visfatin is considered a key molecule in the activation of human leukocytes and the production of pro-inflammatory cytokines; therefore, it seems to be associated with the risk of developing gallstones [119,120]. In this regard, patients with acute cholecystitis often present a higher expression of visfatin in peripheral blood mononuclear cells (PBMCs), serum, and in grossly inflamed gallbladder tissues. Moreover, the gene overexpression of visfatin observed on in vitro models of acute cholecystitis has been frequently accompanied by an increased expression of other pro-inflammatory mediators including IL-10, TNF-α, IL-6, ICAM-1, and VCAM-1 [121]. Similarly, Nien Wang et al. showed that serum visfatin levels were markedly higher in subjects that presented pigment and cholesterol gallstones than in healthy controls. Additionally, serum levels of cholesterol, triglycerides, AST, ALT, leukocyte count, and fasting glucose were significantly higher in those individuals with gallstones. Interestingly, high AST levels and the increased white blood cell count were considered significant predictors of gallbladder lithiasis, while the elevated values of visfatin in serum were also suggested as a significant risk factor for gallstone formation [120]. These results suggested that visfatin could be a predictive marker of inflammation and predisposition to gallbladder lithiasis.

As previously mentioned, evidence accumulated over many years indicates that gallstones can induce an inflammatory microenvironment that increases the risk of developing GBC. However, this risk can be fostered by a greater genetic predisposition [122], for example, the genetic variability present in genes that encode the different ATP-binding cassette (ABC) transporters in the hepatocanalicular membrane, which are involved in the different processes of the exportation of bile salts in biliary tracts, including the transportation of ABCB11, the transport of phosphatidylcholine (ABCB4), and secretion of cholesterol and phytosterols into bile (heterodimer ABCG5/8) [123]. In this regard, a genetic variant in the *ABCG8* gene (variant rs11887534 or D19H) has been associated with a higher gallstone development through cholesterol hypersecretion and cholesterol supersaturation in the bile [122,124]. In addition, other variants (rs1558375, rs17209837, and rs4148808) have been determined in the 7q21.12 region harboring both the *ABCB1* and *ABCB4* genes, which showed a higher risk of developing GBC [28]. A study by Bustos et al. showed that in the Chilean Mapuche ancestry population, variants in *ABCG8* (rs11887534) and *TRAF3* (rs12882491) were associated with GSD. In addition, it was shown that TRAF3 levels were lower in individuals affected by GSD, suggesting that these variants could be used as risk markers for GBC [125,126]. Other mutations occur in the *ABCB4* gene and are classified as nonsense mutations (class I), missense mutations affecting maturation (class II), activity (class III), or protein stability (class IV), and mutations with no identifiable effect (class V) [127]. It has been shown that mutations in this gene increase the risk of developing gallstones in subjects under 40 years, mainly by inducing an *ABCB4* deficiency that results in low biliary phosphatidylcholine concentrations, which is consistent with the spontaneous occurrence of cholecystolithiasis [128,129]. In fact, the homozygous *ABCB4* mutations lead to the complete absence of the phospholipid transporter and no secretion of phospholipids into bile, which finally causes a decrease in the solubility of bile, and consequently, a greater predisposition to bile crystallization and gallstone formation [130]. Other mutations and alterations have been described in different genes and proteins potentially involved in a higher risk of developing gallstones, such as ABCB11 [130], cholesterol 7a-hydroxylase (CYPA1) [131,132], *APOB* gene [133], and cholecystokinin A receptor (CCKAR) [134,135]. In addition, certain alterations in genes related to the immune system, inflammation, and oxidative stress have also been implicated in a greater risk of developing GBC, including mutations in *TLR2, TLR4* [136], *IL1RN, IL1B* [137], *IL10, IL8, IL8RB, RNASEL, VEGF* [138], and *CCR5* [139], as well as rs7504990 variant in the *DCC* [140]. These data show that the predisposition to developing cholecystolithiasis and GBC also have an important genetic background that needs to be considered in GBC carcinogenesis.

Finally, another risk factor associated with GBC is the appearance of bluish and brittle calcifications in the inner gallbladder wall named "porcelain gallbladder" [141]. Porcelain gallbladder has an incidence of less than 1% in patients with gallbladder disease, being more prevalent in women [141]. This rare condition is considered a risk factor for GBC because approximately 60% to 90% of these cases show gallstones [141,142]. Despite the pathophysiology of "porcelain gallbladder" not being clear, this condition could be a consequence of a previous chronic inflammatory process or could be the result of an obstruction produced by gallstones that induce the accumulation and precipitation of calcium in the mucosal layer of the gallbladder wall [143]. Whatever the origin of this disease, it is also unknown whether calcium levels play a role in the “porcelain gallbladder” pathogenesis. Recently, Berger et al. found significantly higher calcium and parathormone (PTH) levels in the plasma of individuals with porcelain gallbladder compared to controls [144], which suggests that individuals with diseases that induce persistent hypercalcemia (e.g., primary hyperparathyroidism) also have a higher risk of developing porcelain gallbladder. In this regard, we venture to propose that persistent hypercalcemia could be an initiating factor of porcelain gallbladder, which would eventually trigger the formation of a chronic inflammatory state in the inner layer of the gallbladder, increasing in this manner the risk of developing GBC. However, more studies are still needed to demonstrate this hypothesis. 

## 5. Infections and Chronic Inflammatory Diseases

### 5.1. Infections Related to Gallbladder Carcinogenesis

Despite gallstone development and genetic abnormalities being important risk factors in inducing GBC, chronic infections also seem to be a factor to consider in gallbladder carcinogenesis [145] since certain pathogens, such as bacteria, viruses, and parasites, are capable of generating direct tissue damage that leads to the activation of an acute or chronic inflammatory response by increasing the levels of different pro-inflammatory cytokines and inducing the reaction of neutrophils and lymphocytes [146].

In the case of bacteria, different bacterial genera have been found in the gallbladder of patients with cholecystitis and cholelithiasis, including *Salmonella* spp., *Escherichia* spp., *Klebsiella* spp., and *Helicobacter* spp. [145]. Another study showed that most bile samples from cholecystectomy patients had *E. coli*, *Salmonella* spp., and *Klebsiella* spp.; however, a few samples also evidenced the presence of *Pseudomonas* sp., *Acinetobacter* sp., *Enterobacter* spp., *Citrobacter freundii*, *Vibrio* spp., and *Serratia marcescens* [147]. In addition, the genetic material of other bacterial species, including *Collibacillus* spp., *Bacteroides fragilis*, *Klebsiella* spp., *Clostridium perfringens*, and *Clostridium* spp., has been found in GBC tissues suggesting that aerobic and anaerobic bacteria can colonize the gallbladder during this malignancy [147].

From these bacterial agents, the most implicated species in gallbladder carcinogenesis are those belonging to the Salmonella genus; a large number of *Salmonella* spp. sequences have been found in GBC samples, suggesting a possible role of the infection by this bacterial genus in GBC carcinogenesis through an inflammatory process [148]. Interestingly, those subjects whose gallbladders had *Salmonella* also showed a higher number of cholecystitis, empyema, and neutrophil infiltration, which indicates the activation of the immune system and inflammatory process [6]. In particular, chronic carriers of *Salmonella typhi* have shown to be implicated in greater development of gallstones [149] and GBC [29,150]. In fact, this risk of acquiring GBC increases about 12-fold in subjects with a history of typhoid fever [29]. This strong likelihood of producing GBC has been associated with the presence of the Vi polysaccharide from *Salmonella typhi* in northern Indian subjects with biliary disease [151]. Evidence has shown that not only chronic infection with *Salmonella typhi,* but also *Salmonella parathypi* presents a high risk of advancing to GBC [150]. On the other hand, a study by Scanu et al. showed that *Salmonella enterica* can induce cell transformation in organoids derived from pre-transformed murine gallbladders from mice deficient in the *Ink4b-Arf-Ink4a* locus that implies an inactive *TP53*, suggesting that alterations in this gene are necessary to malignant transformation [152].

*Helicobacter* has been widely described as inducing different pathologies, specifically *Helicobacter pilori,* which is involved in gastric carcinogenesis [148]. The presence of *Helicobacter* species, such as *Helicobacter bilis* and *Helicobacter pullorum*, has also been demonstrated in bile samples and gallbladder tissues from patients with chronic cholecystitis [153]. In particular, the presence of *Helicobacter bilis* in bile and in biliary tract neoplasms has been linked to a high risk of bile duct carcinoma and gallstones [131,132,133]. Therefore, both *Helicobacter bilis* and *Helicobacter pullorum* have been associated in some way with the risk of developing GBC [23,153,154,155].

Regarding parasitic infections, no direct associations with GBC have been described; however, the presence of liver flukes such as *Clonorchis sinensis, Schistosomiasis japonica* and *Opisthorchis viverrine* have been associated with an increased risk of cholangiocarcinoma [156]. The mechanisms by which these parasites damage the epithelium of the bile ducts are not clear yet, but some authors suggest that trematode eggs cause intense and persistent local inflammation by increasing ROS formation and activating the immune system response [156]. For example, *Clonorchis sinensis* can cause a partial obstruction of the bile ducts, increasing biliary pressure and provoking a repetitive ulceration/inflammation cycle that finally leads to DNA damage and the risk of developing cholangiocarcinoma [157].

Regarding viral infections, some studies performed in Taiwan and China have established that infection with hepatitis B (HBV) and hepatitis C (HCV) viruses increases the risk of extrahepatic bile duct cancer [158,159]. In fact, HBV infection has also been associated with cholangiocarcinoma, cholecystolithiasis, and choledocholithiasis [30]. Interestingly, a study on the Korean population showed a positive association in patients with metabolic syndrome infected with HBV with the risk of bile duct cancer [160]. A meta-analysis by Chen et al. [24] showed that the Epstein–Barr virus (EBV) could have a positive association with the risk of developing hepatobiliary cancers; however, many studies are still needed to determine its effect on the risk of developing GBC.

The development of biliary pathologies from fungal infections is uncommon since these types of infections mainly occur in immunosuppressed patients [161]. An article by Szvalb et al. reported only 42 cases of cholecystitis between 1976 and 2019 where *Candida* infection was involved. About 86% of these cholecystitis cases had *Candida* species as a single microbial agent isolated from gallbladder tissue or fluids, whereas the remaining 14% of cases had one or more co-infecting bacteria. The *Candida* species found were *Candida albicans* (67%), *Candida glabrata* (14%), *Candida parapsilosis* (12%), and *Candida tropicalis* (7%) [162]. Interestingly, a particular fungi genus known as *Fusarium* was also isolated from cholecystitis samples in a neutropenic patient with leukemia who was previously diagnosed with disseminated fusariosis [162]. In addition, a retrospective study that analyzed the bile of patients between 2014 and 2019 who underwent a percutaneous transhepatic cholangiogram (PTC) for obstructive jaundice found that out of 71 patients, only 5 had a positive *Candida* culture (two cases of cholangiocarcinoma and only one of GBC) [17].

All these data indicate that infections could be recognized as important carcinogens that can have a great impact on the development of GBC, since they induce a significant increase in the inflammatory response due to different pathophysiological mechanisms that promote the activation of the immune system, which is considered a determining risk factor in GBC carcinogenesis.

### 5.2. Immune Inflammatory Diseases Involved in Gallbladder Carcinogenesis

Evidence has stated that autoimmune diseases can increase the predisposition to the development of cancer because the recognition made by the immune system of itself as foreign triggers a chronic inflammatory process that leads to carcinogenesis [163,164]. Different autoimmune diseases have been associated with the development of GBC, including celiac disease and Crohn’s disease; however, the most studied GBC-related autoimmune disease is primary sclerosing cholangitis (PSC) [165,166]. PSC is a chronic inflammatory disease characterized by the destruction of the intrahepatic and extrahepatic bile ducts with the consequent fibrosis, which has been described as a high-risk disease for developing cholangiocarcinoma and GBC [167,168,169,170]. Lewis et al. performed an anatomopathological study in 72 gallbladder tissues from PSC patients and found that 69 samples contained at least focal pyloric metaplasia, 27 samples evidenced dysplasia (15 cases with high-grade dysplasia and 12 cases with low-grade dysplasia), and 35 samples showed moderate or advanced diffuse chronic lymphoplasmacytic cholecystitis. In addition, this study also found a significant correlation between dysplasia and the risk of GBC [171]. In another study by Hebillas et al., they showed that of 102 gallbladder cholecystectomy samples from patients with PSC, 8 of them had adenocarcinoma of which 57% were associated with dysplasia [172]. Another study showed that the frequency of gallbladder polyps (GBPs) in patients with PSC was 10.6%, of which 10 had malignant/premalignant lesions, and 4 had high-grade dysplasia [31]. Similarly, a study in Karolinska University Hospital between 1985 and 2006 showed that dysplasia and carcinoma were found in 30% of cases with PSC and a great presence of inflammation and fibrosis [173]. In addition, another study by Castro et al. showed that among 30 autoimmune pathologies, Crohn’s disease and systemic lupus erythematosus (SLE), as well as pernicious anemia and psoriasis, had a higher risk of developing GBC. In the case of SLE, this disease also showed a greater risk of developing extrahepatic bile duct cancer [165]. Therefore, these data suggested that a certain type of autoimmune diseases, such as SLE, Crohn´s disease, and PSC, which are characterized by an abnormal immune reaction to self-antigens, could be an important inflammatory source for the carcinogenic development in GBC. However, further research is required to clarify the mechanisms by which these autoantibodies-released throughout the pathogenesis of these autoimmune diseases-could induce gallbladder neoplasia.

## 6. Consumption of Alcohol and Tobacco as a Risk Factor for Gallbladder Carcinogenesis

Alcohol consumption and smoking are widely known risk factors for some types of malignancies such as colon and lung cancer, respectively [25,32]. However, the role of alcohol and smoking in the development of GBC has not yet been established. For instance, O’Keeffe et al. showed that smoking is associated with a greater GBC risk in both genders; however, alcohol consumption increases the risk of GBC only in men [25], mainly because men consume more alcohol than women [174,175,176]. Alcohol is a well-known inducer of alcoholic fatty liver [177], which is characterized by an increment of cholesterol transportation through the bile canaliculi into the gallbladder, inducing cholesterol crystallization and thus gallstone formation. In addition, greater alcohol consumption can lead to liver cirrhosis, which is directly linked to a higher incidence of gallstones [178,179]. Another study by McGee et al. analyzed 26 prospective studies in which the risk of bile duct cancer was evaluated in people who consumed alcohol and smoked. Results showed that smoking increased the risk of developing intrahepatic bile duct, extrahepatic bile duct, and ampulla of Vater cancers, but not GBC. In addition, alcohol consumption was also associated with a higher risk for developing intrahepatic bile duct cancer, but not for GBC [16]. An article by Wenbin et al. reviewed 10 case–control studies and one prospective study, showing that smoking was able to augment the risk of developing GBC, being an independent factor from alcohol consumption and gallstone formation for GBC development [14].

## 7. Exposure to Aflatoxins and Other Elements and Compounds as Risk Factors for Gallbladder Carcinogenesis

Aflatoxins are compounds derived from some fungi species, such as *Aspergillus flavus* and *Aspergillus parasiticus*, which can be found in certain foods such as corn or peanuts [180]. Aflatoxins form adducts with albumin from the blood, binding in lysine residues [181]. Some authors have estimated that the consumption of aflatoxins could increase the risk of GBC and liver cancer [182,183] since this compound could be accumulated in the bile ducts [184,185]. A study carried out in Chile evaluated the effect of the weekly consumption of *ají rojo* (a type of hot dried pepper) in patients with GBC, finding that 64% of these cases had AFB1 adducts in plasma [186]. Another study by Koshiol et al. showed that the plasma aflatoxin B_1_ (AFB_1_) was present in 32% of GBC patients. Interestingly, none of the 54 sequenced tumors had the R249S mutation in the *TP53* gene, which has been associated with aflatoxin exposure [187], suggesting that there could be other mechanisms involved in gallbladder carcinogenesis which are different from those observed in the development of other cancer types.

GBC may also be induced by exposure to certain toxic elements and compounds. For instance, arsenic (As) is a highly toxic semi-metallic element that can induce disease in humans by different sources of exposure, mainly via contaminated groundwater, residues from textile manufacturing, pesticides, mine dust and others, representing one of the greatest threats to public health in different regions of the world [188]. It has been described that the mechanisms by which arsenic can induce cancer are different, including inducing ROS formation and modulating epigenetic changes through histone modification and DNA methylation [189]. This element can enter the body predominantly by consuming contaminated water used for drinking, for food preparation, and for vegetable irrigation, as well as by exposure to pesticides and other chemicals products [188]. Exposure to the trivalent inorganic form (iAs) (III) and its mono- and dimethylated derivatives MMA(III) and DMA(III), respectively, are associated with cancers of the skin, lung, bladder, kidney, and liver [189,190]. Interestingly, in certain locations a significant correlation has been observed between the frequency of GBC and the presence of arsenic in waters destined for drinking or food preparation, suggesting a potential carcinogenic role in gallbladder carcinogenesis [191]. Surprisingly, some studies have suggested arsenic’s protective effect on GBC [192]. In this regard, Barahona et al. found a protective effect of iAs% on the risk of GBC (OR = 0.80, *p* = 0.03), showing that a poor metabolizing capacity, marked by the highest percentage of MMA, showed a protective effect (OR = 0.85, *p* = 0.08). On the other hand, the highest percentage of DMA, a marker of efficient arsenic metabolism, showed a non-protective effect on the risk of GBC (OR=1.10, *p* = 0.06). Interestingly, the presence of some variants of AS3MT (rs9527) and FTCD (rs61735836) were shown to be associated with MMA, iAS, and a low risk of GBC. While the rs11191527 variant of the AS3MT gene showed discrepant results [35].

Other elements and compounds such as nickel (Ni), cadmium (Cd), chromium (Cr) [33,193], molybdenum (Mo) [194], copper (Cu) [195], asbestos [34], coal or wood dust [196], and radon (Rn) have been associated with GBC or bile duct cancer [197]. For example, serum Mo levels are higher in patients with gallstones and GBC [194], suggesting that exposure to this metal could be a risk factor for developing GBC. By contrast, lower serum levels of selenium (Se), zinc (Zn), manganese (Mn), vitamin E, and vitamin C, elements that have been described as antioxidant agents and modulators of the immune system [198], were found in GBC when compared to cholelithiasis and healthy controls. In fact, copper (Cu) levels and the Cu/Zn ratio also showed a significant increase in the serum, bile, and gallbladder tissues of subjects with GBC compared to the other elements [195], indicating that the deficiency of Se, Zn, Mn, vitamin E, and vitamin C could be a risk for developing GBC, while the Cu/Zn ratio could be another biological parameter to also determine the risk for GBC.

Different articles have addressed gallbladder carcinogenesis in different ways, focusing mainly on epidemiological [67,80,199,200] and clinical aspects [201]. This review comprehensively addresses the cellular and/or molecular aspects induced by risk factors that lead to gallbladder carcinogenesis. These aspects include immune system activation, chronic inflammation, nutritional features, infections, genomic alterations/genetic variability, signaling pathway activation, and lipid metabolism. Many of these mechanisms result in gallstone formation and/or chronic inflammation that subsequently leads to GBC. Moreover, we address recent topics regarding neoantigens that could be useful for developing tumor immunotherapies against GBC and the implications of aflatoxin consumption from food that initiate an inflammatory state before gallbladder carcinogenesis. In summary, this article emphasizes GBC from the many aspects involving an inflammation response that could help to better understand this devastating disease.

**Figure 3:**
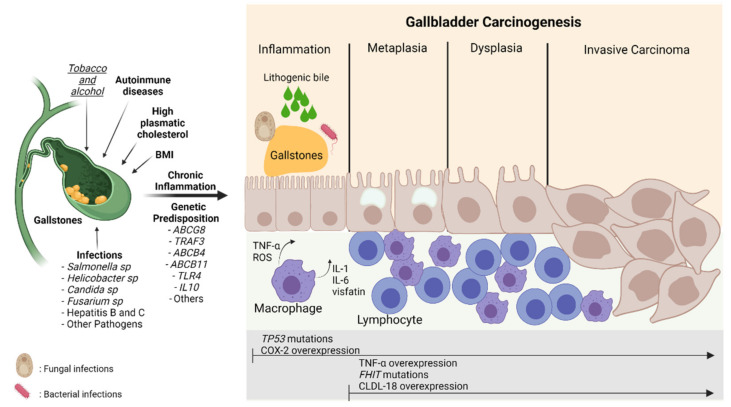
Carcinogenesis process in gallbladder cancer. The damage caused by the presence of risk factors such as gallstones, infections, lithogenic bile, alcohol, smoking, and genetic predisposition can induce continuous damage in the mucosa of the gallbladder, which is characterized by a chronic inflammatory state mainly highlighted by the activation of macrophages and lymphocytes that leads to the release of pro-inflammatory cytokines (TNF-α, IL-6, IL-1) and ROS stimulating the carcinogenic metaplasia/hyperplasia–dysplasia–carcinoma transition. This process can be marked by different gene alterations and protein expressions such as *TP53* and *FHIT* mutations and COX-2, TNF-α, and CLDN-18 overexpression, respectively. BMI: Body Mass Index; TNF-α: Tumor necrosis factor-alpha; ROS: Reactive oxygen species; IL-1: Interleukin-1; IL-6; Interleukin-6; CLDN-18: Claudin 18; COX-2: Cyclooxygenase 2; TP53: Tumor protein 53; FHIT: Fragile Histidine Triad Diadenosine Triphosphatase. The risk factors in bold mean strong evidence. The risk factor in italics and underlined means weak evidence.

Figure 3 shows the relationship between the risk factors described above and their involvement in GBC inflammation and carcinogenesis.

## 8. Conclusions

The cumulative evidence described in this review shows that GBC is a chronic inflammatory disease promoted by different risk factors including gallstone disease (GSD), sedentary lifestyle, smoking, alcohol consumption, metabolic disorders, high-fat diet, hypercholesterolemia, and some types of infections. In addition, gallbladder carcinogenesis has been strongly associated with specific populations (e.g., Mapuche ancestry), possibly because these populations present some genetic background (e.g., *ABCG8* and *TRAF3* gene variants) that predispose them to being more susceptible to gallstone formation and hence a greater predisposition to the development GBC, which could explain the high incidence of GBC in this population.

On the other hand, there are certain genetic alterations that could induce or maintain a gallbladder carcinogenic state. For instance, *TP53* mutations are usually described as regulators of the immune response through the NF-κB pathway, suggesting that alterations in *TP53* could be considered a pivotal step to gallbladder carcinogenesis. In addition, mutations in other genes (e.g., *FHIT*, *IL10*, and *IL8*), as well as the overexpression of many inflammatory proteins (e.g., COX-2 and TNF-α), are usually considered crucial in the maintenance of the chronic inflammatory state characterized by a continuous damage/regeneration loop that results in the carcinogenic process. Therefore, regarding the above-mentioned topic, that is, whether populations that already have a greater inherited genetic susceptibility (e.g., Mapuche ancestry) are able to develop additional genetic alterations, the incidence of GBC might be eventually maintained or increased; thus, the search for new therapies that seek to avoid chronic inflammation and continuous carcinogenic development in the affected population would have a great impact in decreasing the incidence of this malignancy.

Infections by different pathogens (e.g., bacteria) can also generate an exacerbated immune response due to the repetitive damage produced in the gallbladder mucosa through the different molecular mechanisms that these pathogens use to survive in the host. This review has shown that the presence of different bacterial genera (e.g., *Salmonella spp* and *Helicobacter* species) in the bile of patients with cholecystitis is strongly associated with gallbladder carcinogenesis, suggesting that infections by different pathogens could be a feasible cause of a chronic inflammation state to promote GBC [26,202,203].

Similarly, autoimmune diseases also predispose to a chronic inflammatory state in the affected tissues. One of these pathologies is PSC, which is characterized mainly by the destruction of the intrahepatic and extrahepatic bile ducts, which induces a greater presence of inflammatory components, suggesting that PSC and other inflammatory diseases could be significant to GBC development in these patients. The treatments for such diseases are mainly focused on reducing the inflammatory effect and the activity of the immune system in order to slow down the pathological consequences in patients, but not on completely eliminating the chronicity of the tissue damage. Furthermore, if inherited and acquired mutational heterogeneity is added to the previous variables, the scenario is very favorable to promoting a persistent carcinogenic development.

Smoking and alcohol consumption are widely known risk factors for several cancer types; however, these factors have not been well defined for GBC. This article reviewed some studies that propose smoking could be a risk factor for the development of GBC. Regarding the involvement of alcohol in GBC, the evidence is not conclusive because studies have not found a reliable correlation between GBC and the amount of alcohol consumed by individuals. Interestingly, some studies have found that men have a higher risk of developing GBC than women. This could be explained by the fact that alcohol consumption is frequently higher in men than in women. However, in recent years a considerable increase in smoking and alcohol consumption has been evidenced among young people; therefore, it would not be unusual to observe an increase in the GBC incidence due to these causes in the coming years. Finally, there is worldwide concern about human exposure to harmful elements and compounds (e.g., arsenic) commonly found in water sources and foods. Unfortunately, this problem is more frequent in lower-income countries where health policies may be insufficient compared to developed countries. Although some of these elements have not been directly associated with GBC yet, they could be an added value in the predisposition to the development of GBC. An example of this is arsenic, an element widely described as dangerous to humans. Regarding this, some studies have shown arsenic as a protective element and others as a risk factor for GBC; therefore, considering this problem, the "protective" concept is still inconclusive, requiring even more studies to demonstrate this hypothesis.

In summary, gallbladder carcinogenesis is induced by several risk factors that regardless of their origin trigger a chronic inflammatory state. Therefore, one of the challenges for the scientific community is the search for preventive treatments that can reduce the effect of chronic inflammation in the gallbladder, and hence, slow the onset of GBC. Finally, it is necessary that government authorities along with researchers commit to promoting massive sanitary and health prevention strategies in the global population, especially in poor and developing nations.
